# Integrated Optoelectronic Position Sensor for Scanning Micromirrors

**DOI:** 10.3390/s18040982

**Published:** 2018-03-26

**Authors:** Xiang Cheng, Xinglin Sun, Yan Liu, Lijun Zhu, Xiaoyang Zhang, Liang Zhou, Huikai Xie

**Affiliations:** 1 School of Aerospace Engineering, Xiamen University, Xiamen 361005, China; 19920161151425@stu.xmu.edu.cn (X.S.); 19920150150658@stu.xmu.edu.cn (Y.L.); 35120171150981@stu.xmu.edu.cn (L.Z.); 2 Department of Electrical and Computer Engineering, University of Florida, Gainesville, FL 32611, USA; xzhang292@ufl.edu (X.Z.); l.zhou@ufl.edu (L.Z.); hkxie@ece.ufl.edu (H.X.)

**Keywords:** QPD, MOEMS, position sensor, optoelectronic position sensor, MEMS mirror, micromirror

## Abstract

Scanning micromirrors have been used in a wide range of areas, but many of them do not have position sensing built in, which significantly limits their application space. This paper reports an integrated optoelectronic position sensor (iOE-PS) that can measure the linear displacement and tilting angle of electrothermal MEMS (Micro-electromechanical Systems) scanning mirrors. The iOE-PS integrates a laser diode and its driving circuits, a quadrant photo-detector (QPD) and its readout circuits, and a band-gap reference all on a single chip, and it has been fabricated in a standard 0.5 μm CMOS (Complementary Metal Oxide Semiconductor) process. The footprint of the iOE-PS chip is 5 mm × 5 mm. Each quadrant of the QPD has a photosensitive area of 500 µm × 500 µm and the spacing between adjacent quadrants is 500 μm. The iOE-PS chip is simply packaged underneath of an electrothermally-actuated MEMS mirror. Experimental results show that the iOE-PS has a linear response when the MEMS mirror plate moves vertically between 2.0 mm and 3.0 mm over the iOE-PS chip or scans from −5 to +5°. Such MEMS scanning mirrors integrated with the iOE-PS can greatly reduce the complexity and cost of the MEMS mirrors-enabled modules and systems.

## 1. Introduction

Micro-opto-electro-mechanical systems (MOEMS) are microsystems based on micro-optical devices fabricated using micromachining [1,2,3]. The most important MOEMS devices are scanning micromirrors that can perform tip-tilt angular scanning or piston (phase-only) scanning or tip-tilt-piston (TTP) scanning [4,5]. Scanning micromirrors have been widely used in biomedical imaging [6], spectroscopy [7], displays [8], optical communication [9], and so on. For any of these applications, it is especially necessary to monitor the vertical piston displacement and/or the tip-tilt angle of the mirror plate of the micromirror employed. The scanning can be generated by electrostatic, electrothermal, electromagnetic, or piezoelectric actuation [10]. Among these actuation mechanisms, electrothermal actuation based on bimorphs or multimorphs provides unique advantages in terms of achieving large scan range, large aperture, high fill factor and low driving voltage simultaneously [11]. The most commonly used methods of realizing position sensing of mirror plates include capacitive and piezoresistive sensing [12]. However, these two methods cannot be applied to electrothermal bimorph based micromirrors due to the large displacement and the absence of silicon or polysilicon in the bimorphs/multimorphs. Thus, an inductive position sensing method has been developed particularly for an electrothermal TTP micromirror that can move vertically by more than 100 μm and achieve a tilt angle sensing resolution of 0.0013° in both lateral axes [13], but the miniaturization of the coils especially for simultaneous tip, tilt and piston sensing is quite challenging. Optical TTP position sensing was also proposed [14,15], but the packaging size was relatively large due to the need of assembling multiple photodetectors. Therefore, an integrated optoelectronic position sensor (iOE-PS) was proposed by the authors for more compact integration [16].

In this work, the iOE-PS has been designed and fabricated, and it has been successfully applied to measure the linear displacement and tilting angle of an electrothermally-actuated MEMS scanning mirror. This paper is organized as follows. In Section 2, the MEMS mirror is briefly introduced and the proposed design of MEMS position sensing based on the iOE-PS is described. In Section 3, the optical system of the MEMS/iOE-PS module is analyzed, the QPD design and readout circuit design are given, and the quasi-static response of the position sensing is simulated to determine the optimal working distances between the mirror plate of the MEMS mirror and the surface of the iOE-PS chip. In Section 4, experimental results are presented.

## 2. The MEMS Mirror and the Position Sensing Principle

**Figure 1:**
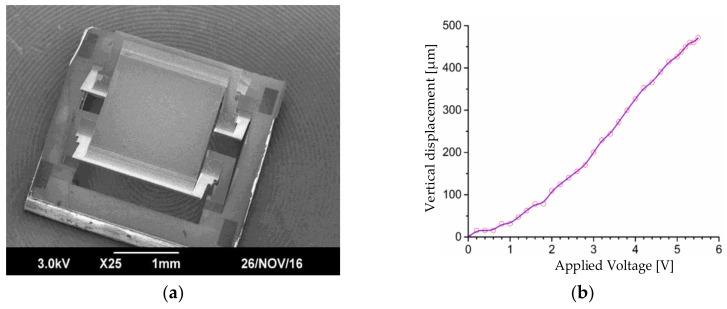
The MEMS micromirror under investigation. (**a**) SEM of the micromirror; (**b**) The measured vertical displacement vs. applied voltage.

The MEMS micromirror under investigation is shown in Figure 1. This is a micromirror with 1-axis angular scanning plus large piston. The footprint of the MEMS device is 3 × 3 mm^2^ and the size of the mirror plate is 2 × 2 mm^2^. The actuator structure is made of three bimorphs (Al/SiO^2^) and two rigid frames (Si supported) with properly chosen lengths of the five segments for lateral shift and tilting compensation, or so called the lateral-shift-free, large-vertical-displacement (LSF-LVD) actuator [17]. A Pt heater is embedded between Al and SiO_2_ for electrothermal actuation. The central mirror plate is supported by two sets of LSF-LVD actuators on its two opposing sides. When a same voltage is applied to the two actuators, the mirror plate moves vertically; when two different voltages are applied to the two actuators, the mirror plate rotates. The vertical scanning displacement is the range of 500 μm and the tilting angle range is about 20°.

**Figure 2:**
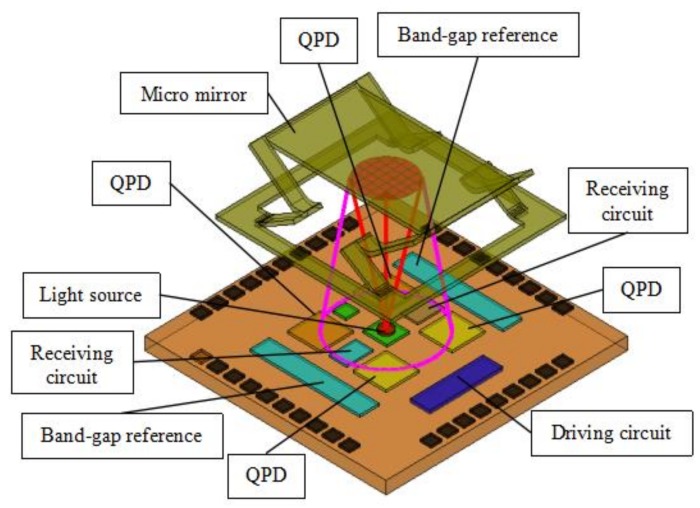
Schematic of the micromirror and integrated optoelectronic position sensor (iOE-PS) module.

From Figure 1, it is worth noting that it is hollow under the mirror plate, which is critical for the proposed method to work. It is proposed that the iOE-PS is placed directly under the mirror plate, as illustrated in Figure 2. The iOE-PS consists of two parts: a transmitter and a receiver. The transmitter is basically a light source such as a vertical cavity surface emitting laser (VCSEL) while the receiver is a monolithic QPD. The QPD, the readout circuit for the QPD, and the driver circuit for the VCSEL are all integrated on the iOE-PS chip. The VCSEL chip is directly bonded on the iOE-PS chip. So, all the devices and circuits are integrated in a single compact unit. Upon operation, the light emitted from the VCSEL is reflected by the mirror plate and is then received by the QPD that generates four photocurrents. The photocurrents are used to calculate the displacement and tilting angle of the mirror plate. The needed reference voltage source and constant current source are provided by the band-gap reference also integrated on the iOE-PS chip.

## 3. The Detailed Design of the MEMS/iOE-PS Module

### 3.1. Optical Design and Analysis

**Figure 3:**
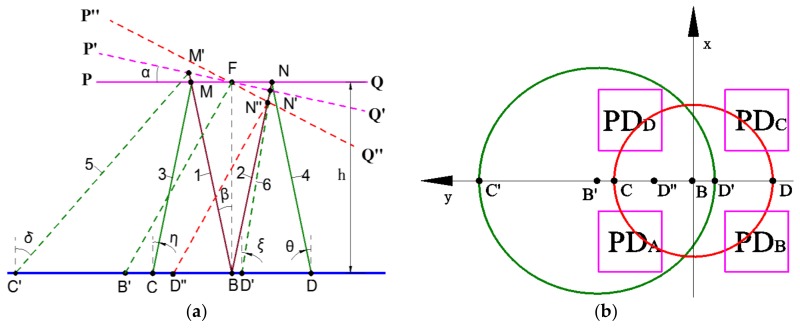
Optical theoretical model. (**a**) Optical path diagram of the reflected light at different mirror plate positions; (**b**) Schematic diagram showing the locations of the PDs and the light spots at different mirror plate positions.

We will first look at the optical system design and find out how the PD (Photodetector) size and the distance from the PD to the mirror plate will affect the responsivity and bandwidth of the MEMS/iOE-PS system. Optical theoretical model is shown in Figure 3, the optical path diagram of the iOE-PS/MEMS module is shown in Figure 3a, where B is the center of the light source and *β* is the beam divergence half-angle. The general range of *β* is 10~120°, its typical values for surface-emitting laser or LED are 20°, 30°, and 90°. Assume the plate is at the horizontal position (PQ), the edge rays 1 and 2 reach the points M and N on the mirror plate and are reflected back as the rays 3 and 4 to the points C and D on the surface of the iOE-PS chip, respectively. These two rays’ incident angles to the chip are *η* and *θ*, respectively, which are both equal to *β*. When the mirror plate rotates by *α* to the position of P’Q’, the original rays 1 and 2 reach the points M and N’ on the mirror plate, and then the corresponding reflected rays 5 and 6 reach the points C’ and D’ with the incident angles of *δ* and *ξ*, respectively. It can be seen from Figure 3a that *δ* = 2*α* + *β* and *ξ*= *β* − 2*α*. The center ray of the light source is incident on the point F, and then is reflected to the point B’, which is located between the points C’ and D’ If 2*α* > *β*, D’ will be located in the left side of B and *ξ* becomes 2*α* − *β*, which is D’’ in Figure 3a. The positions of these points can be obtained using Equations (1)–(4).

(1) $$\frac{{\mathrm{BM}}^{\prime }}{\sin∠{\mathrm{BFM}}^{\prime }}=\frac{\mathrm{BF}}{\sin∠{\mathrm{BM}}^{\prime }F}=\frac{h}{\sin(\frac{\pi }{2}-\alpha -\beta )},$$

When 2*α* < *β*,

(2) $${BD}^{\prime }=\frac{h\cos\alpha }{\cos(\alpha -\beta )}[\sin\beta +\cos\beta \tan(\beta -2\alpha )],$$

(3) $${BC}^{\prime }={BM}^{\prime }\sin\beta +{BM}^{\prime }\cos\beta \tan\delta =\frac{h\cos\alpha }{\cos(\alpha +\beta )}[\sin\beta +\cos\beta \tan(\beta +2\alpha )],$$

When 2*α* > *β*,

(4) $${BD}^{\prime \prime }=\frac{h\cos\alpha }{\cos(\alpha -\beta )}[\cos\beta \tan(2\alpha -\beta )-\sin\beta ],$$

**Figure 4:**
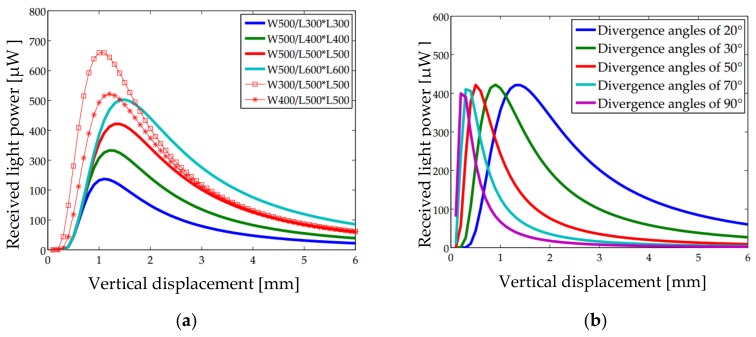
Received light power versus vertical displacement. (**a**) Different *W*&*L* with the divergence angle of 20°; (**b**) Different divergence angles with *W* of 500 μm and *L* of 500 μm.

When the mirror plate is at the position of PQ in Figure 3a, the reflected light on the chip possesses the same Gaussian distribution as that emitted from the light source [18,19]. Figure 3b shows the schematic diagram of the locations of the four PDs of the QPD and the illumination areas at two different mirror plate positions PQ and P’Q’, where the red circle is for PQ and the green circle is for P’Q’. The origin of the coordinate is set at B. The optical intensity at (*x*, *y*), *I*(*x*, *y*), is given by Equation (5). Then the optical power received by each PD can be obtained by Equation (6).

(5) $$I(x,y)=\frac{P_{0}}{2\pi \sigma^{2}}\exp\{-\frac{x^{2}+y^{2}}{2\sigma^{2}}\},$$

(6) $$P_{A}=P_{B}=P_{C}=P_{D}=\iint_{S_{A}}I(x,y)dxdy=\int_{\frac{W}{2}}^{\frac{W}{2}+L}\int_{\frac{W}{2}}^{\frac{W}{2}+L}\frac{P_{0}}{2\pi \sigma^{2}}\exp\{-\frac{x^{2}+y^{2}}{2\sigma^{{}^{2}}}\}dxdy,$$

where *P*_0_ is the total optical power of the VCSEL. For the VCSEL employed in this study, the wavelength is 850 nm and the power is 1.8 mW. In Equation (6), *σ* (*σ* = 2*h*tan*β*) is the radius of the light spot on the MEMS mirror plate, W is the PD spacing, and L is the length of a single PD. Plugging *σ* = 2*h*tan*β* into Equation (6) yields Equation (7). Here, the sum of the light powers received by the four PD quadrants can be taken as the output of the QPD when the micromirror plate is horizontal. A preliminary simulation of the total receiving light power over h is shown in Figure 4.

(7) $$P=P_{A}+P_{B}+P_{C}+P_{D}=4\iint_{S_{A}}I(x,y)dxdy=4\int_{\frac{W}{2}}^{\frac{W}{2}+L}\int_{\frac{W}{2}}^{\frac{W}{2}+L}\frac{P_{0}}{8\pi h^{2}\tan^{2}\frac{\beta }{2}}\exp\{-\frac{x^{2}+y^{2}}{8h^{2}\tan^{2}\frac{\beta }{2}}\}dxdy$$

As shown in Figure 4, there is a maximum point in each curve, forming two monotonic changing regions: an upward region and a downward region. At a small distance, as the light is concentrated in the center, the output is proportional to the received light spot. The downward region mainly because the light density attenuates during the gradual saturation of the illuminance area. The upward region has a larger slope, i.e., this region is more sensitive to the change of the displacement, which is preferred for position sensing. In addition, the vertical displacement *h* corresponding to the maximum output increases with the increase of *W*, the decrease of *L*, and the decrease of *β*. Among them, the monotonically changing range is the most significantly affected by *β* and is less influenced by *W* and *L* shown in Figure 4a. However, the maximum output changes with *W* and *L* considerably; a better resolution can be obtained at a larger *L* and a smaller *W*.

**Figure 5:**
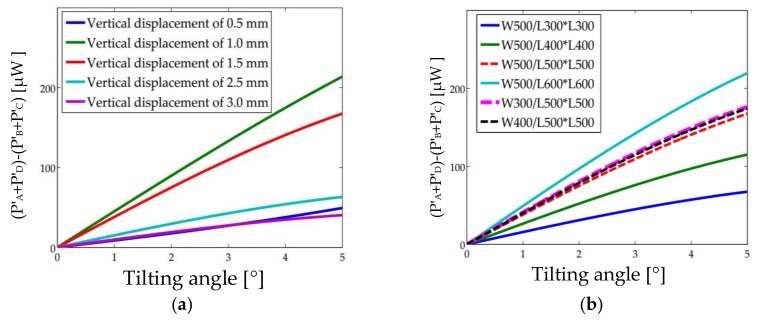
Output versus tilting angle. (**a**) Different vertical displacements with 2*β* of 20°, *W* of 500 μm, and *L* of 500 μm; (**b**) Different *W* and *L* with *β* = 20° and *h* = 1.5 mm.

When the MEMS mirror plate tilts at an angle *α*, the center ray out of the VCSEL is reflected back to point B’(*y*_0_, 0), where *y*_0_ is equal to B’B in Figure 3b. The light intensity at (*x*, *y*) with a tilting angle *α* is given by Equation (8). The light powers received by the four quadrants of the QPD are given by Equations (9) and (10), where *y*_0_ and *σ’* can be obtained by Equations (12) and (13). The *α* can be obtained via *P*_α_, which is given by Equation (11). The relation of *P*_α_ over *α* under different vertical displacements and PD arrangements are depicted in Figure 5. The *P_α_*-*α* curves at different displacements always show a good linearity in the range of 5° when 2*α* < *β*; In Figure 5a, the slope is the largest for the *h* = 1.0 mm, meaning the angular sensitivity is the highest around *h* = 1.0 mm. In Figure 5b, for a fixed vertical displacement *h* = 1.5 mm, the slope (i.e., the angular sensitivity) increases with the increase of *W* and the decrease of *L* but with a higher dependence on *L*. Therefore, a proper combination of *W* and *L* needs to be found to achieve high sensitivity to both *α* and *h*. Based on Equations (8)–(13), *W* and *L* are chosen as 500 μm and 500 μm after multiple iterations.

(8) $$I^{\prime }(x,y)=\frac{P_{0}}{2\pi {\sigma^{\prime }}^{2}}\exp\{-\frac{x^{2}+{\left(y-y_{0}\right)}^{2}}{2\sigma^{2}}\},$$

(9) $${P^{\prime }}_{A}={P^{\prime }}_{D}=\iint_{S^{\prime }}I^{\prime }(x,y)dxdy=\int_{\frac{W}{2}}^{\frac{W}{2}+L}\int_{\frac{W}{2}}^{\frac{W}{2}+L}\frac{P_{0}}{2\pi {\sigma^{\prime }}^{2}}\exp\{-\frac{x^{2}+{\left(y-y_{0}\right)}^{2}}{2{\sigma^{\prime }}^{2}}\}dxdy,$$

(10) $${P^{\prime }}_{B}={P^{\prime }}_{C}=\iint_{S^{\prime }}I^{\prime }(x,y)dxdy=\int_{-\frac{W}{2}}^{-(\frac{W}{2}+L)}\int_{-\frac{W}{2}}^{-(\frac{W}{2}+L)}\frac{P_{0}}{2\pi {\sigma^{\prime }}^{2}}\exp\{-\frac{x^{2}+{\left(y-y_{0}\right)}^{2}}{2{\sigma^{\prime }}^{2}}\}dxdy,$$

(11) $$P_{\alpha }=({P^{\prime }}_{A}+{P^{\prime }}_{D})-({P^{\prime }}_{B}+{P^{\prime }}_{C}),$$

(12) $$y_{0}=\frac{1}{2}({BC}^{\prime }-{BD}^{\prime })=\frac{1}{2}h\cos\alpha [\frac{\sin\beta +\cos\beta \tan(2\alpha +\beta )}{\cos(\alpha +\beta )}-\frac{\sin\beta +\cos\beta \tan(\beta -2\alpha )}{\cos(\alpha -\beta )}],$$

(13) $$\sigma^{\prime }=\frac{1}{2}({BC}^{\prime }+{BD}^{\prime })=\frac{1}{2}h\cos\alpha [\frac{\sin\beta +\cos\beta \tan(2\alpha +\beta )}{\cos(\alpha +\beta )}+\frac{\sin\beta +\cos\beta \tan(\beta -2\alpha )}{\cos(\alpha -\beta )}],$$

### 3.2. PD Design

In our previous work [20], a multi-finger PIN PD was designed and optimized in a standard 0.25 µm BCD process. Another PD with the size of 500 × 500 µm^2^ and the structure of n+/N-well/P-sub was optimized on a standard 0.5 µm CMOS process [21]. Through these efforts, PDs with large dynamic range, high linearity, low dark current, high sensitivity and stable operation have been demonstrated. In this work, the proposed QPD is formed with four same PDs that are based on the previous PD designs.

**Figure 6:**
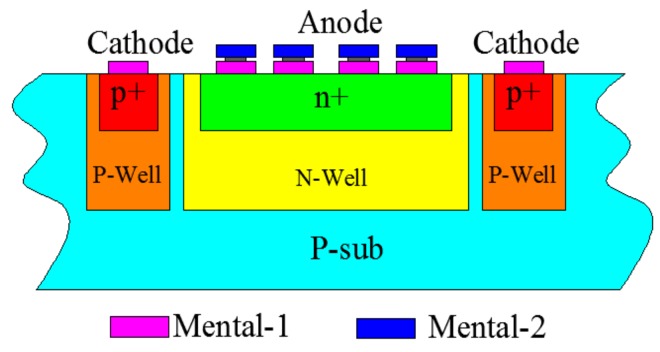
Schematic of the n+/N-Well/P-sub photo-detector.

**Figure 7:**
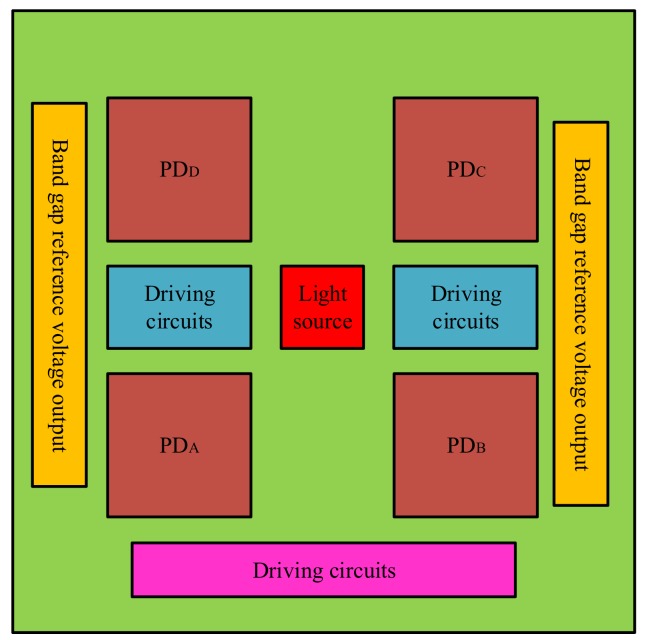
The architecture of the iOE-PS circuit.

Each PD of the QPD has a photosensitive area of 500 × 500 µm^2^ and a n+/N-Well/P-sub structure (shown in Figure 6) on a CSMC 0.5 µm CMOS process with no process modification [22], its position in the iOE-PS is shown in Figure 7. P-type wafers with a doping concentration near 10^15^ cm^−3^ is used as the substrate. By ion implantation, the N-well and P-well are formed and used for both CMOS ICs and PDs. The PDs formed by the N-well/P-sub junction. The n+/N-well high-low doping junction structure is added to reduce surface recombination and improve the responsivity in the longer wavelength range of 650~1000 nm, compared to the usual N-well/P-sub PDs. For example, an 850 nm laser is used in this work, whose responsivity reached 0.3 A/W. Meanwhile, it was measured that the frequency of the QPD could reach up to 1 MHz, which fully meets the requirement of the micromirror testing, where the typical frequency range is from 1 Hz to 10 kHz.

### 3.3. Readout Circuit Design

**Figure 8:**
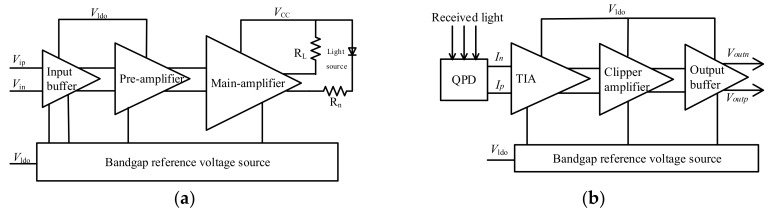
The block diagrams of the driving circuits. (**a**) The driving circuits; (**b**) The receiving circuits.

The architecture of the iOE-PS circuit is shown in Figure 7. The iOE-PS circuits are simulated with the light source and PDs in Cadence [23]. The block diagram of the driving circuits is shown in Figure 8a, where the gains of the pre-amplifier and main-amplifier are 10.3 dBΩ and 15.8 dBΩ, respectively. So the overall gain of the driving circuits has reached up to 26.1 dBΩ, which can provide a maximum bias current of 27 mA. As for the receiving circuit shown in Figure 8b, it is composed of a bandgap reference voltage source, a transimpedance amplifier (TIA), a three-stage cascaded clipper amplifier, and an output buffer. The total gain of the receiving circuits is 88.1 dBΩ with a power supply of 5 V, and its f_-3dB_ is about 3.5 MHz. The equivalent input noise current is 10 pA/$\sqrt{\mathrm{Hz}}$. Under the influence of the simulated QPD current of 0~6 μA, the dynamic swing of the output voltage reaches 94 mV, and the linearity is good.

### 3.4. Optical Simulation

**Figure 9:**
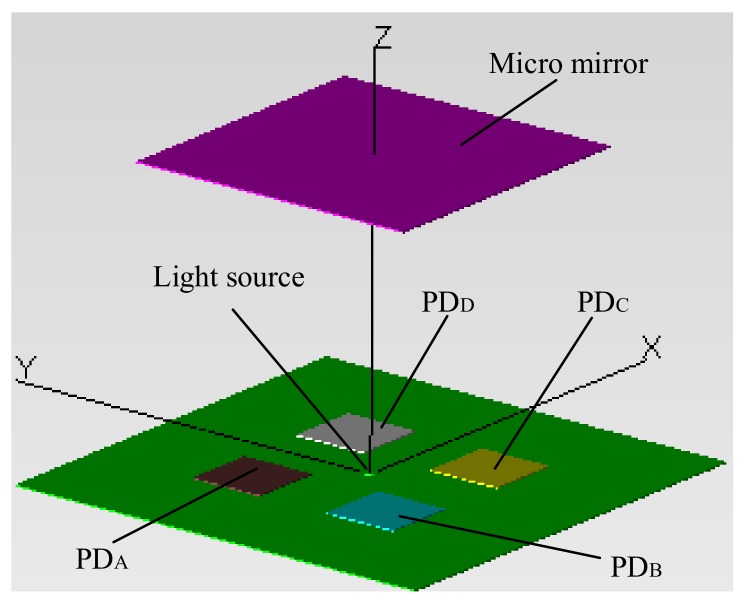
Model of the optical system.

In order to get more practical output characteristics, optical simulation of the system is necessary, which can be done with TracePro [24]. The model of the optical system is shown in Figure 9. A 650 nm RCLED with the divergence angle of 90° and an 850 nm VCSEL with the divergence angle of 20° are chosen as the light sources. The optical power on each PD can be obtained directly by ray tracing. The typical titling angle of an angular scanning micromirror is about 10°. As discussed in Section 3.1, the PD spacing *W* is set as 500 μm. What is the most important is that the optimal ranges of *L* and *h* need to be determined. 

#### 3.4.1. Vertical Displacement Simulation

**Figure 10:**
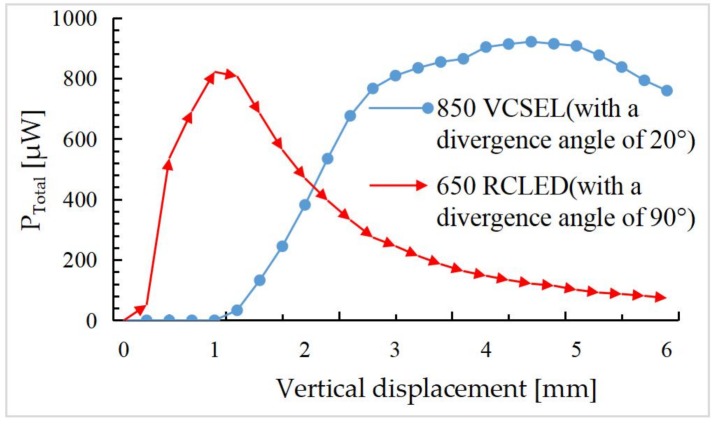
The total received light power versus the vertical position of the mirror plate.

As discussed in Section 3.1, the vertical displacement is measured by detecting the change of the total flux from all four PDs. First, the titling angle is set to 0° and the mirror plate is moved vertically from the iOE-PS chip to the position of 6 mm above the chip. *P*_Total_, the total optical power of the QPD, is gained by ray tracing and plotted at a step interval of 0.25 mm in Figure 10 where two light sources with different divergence angles are considered.

It can be seen that *P*_Total_ reaches its maximum when the micromirror displacement is 1.2 mm for the 90° RCLED or 4.5 mm for the 20° VCSEL. For the 20° VCSEL, there is a large linear range of 1.3–3.0 mm. It is worth noting that a near zero *P*_Total_ in 0–1.0 mm is obtained for the fact that almost all of the reflected light is incident in the spacing between the PDs when the mirror plate is too close to the chip and the divergence angle of the light is small. For the 90° RCLED, the response in the small displacement (0.25–0.75 mm) range is large, but the slope becomes nearly zero at about 1.0 mm. In this work, for the demonstration purpose, the small-divergence VCSEL is chosen for its larger linear range.

#### 3.4.2. Tilting Angle Simulation

**Figure 11:**
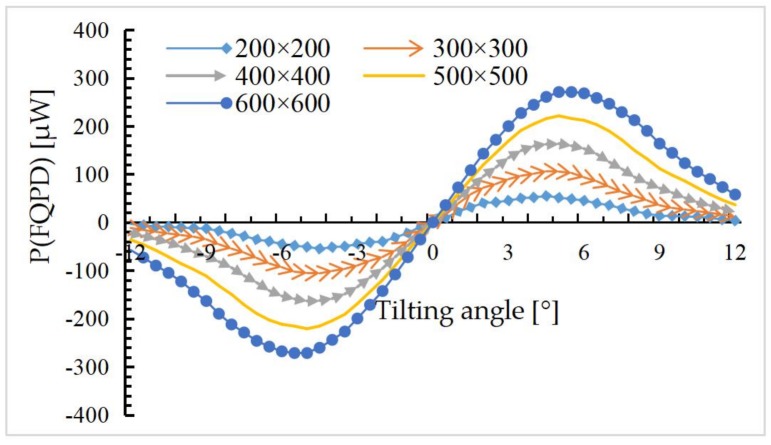
*P_α_* over *α* for different PD areas at *h* = 2 mm.

According to the result shown in Figure 10, the distance between the iOE-PS chip and the mirror plate is set to 2 mm (in the linear region). The PD area can be set to different sizes, such as 200 × 200 μm^2^, 300 × 300 μm^2^, 400 × 400 μm^2^, 500 × 500 μm^2^, and 600 × 600 μm^2^ respectively. Meanwhile, the mirror plate is rotated from −12 to 12° at a 0.5° interval. According to Equation (11), *P_α_* over *α* for different PD areas is plotted in Figure 11, where the vertical displacement of the mirror plate *h* is set at 2 mm.

It can be seen that the *P_α_* over *α* curves show a monotonically-increasing range and larger PDs have slightly larger monotonic increase range. After the PD area of 500 × 500 μm^2^, the increase becomes very small. So in order to minimize the overall chip size the PD area is set as 500 × 500 μm^2^. 

Based on above analyses, the four PDs of the QPD are designed with the same photosensitive area of 500 × 500 μm^2^, and the PD spacings are all 500 μm. The mirror plate of the MEMS mirror is placed over the iOE-PS chip with the initial distance set at about 2 mm. Then it is expected that the iOE-PS can monitor the vertical piston displacement of about ±1mm and the tilting angle of ±5°.

## 4. Experiments and Discussion

**Figure 12:**
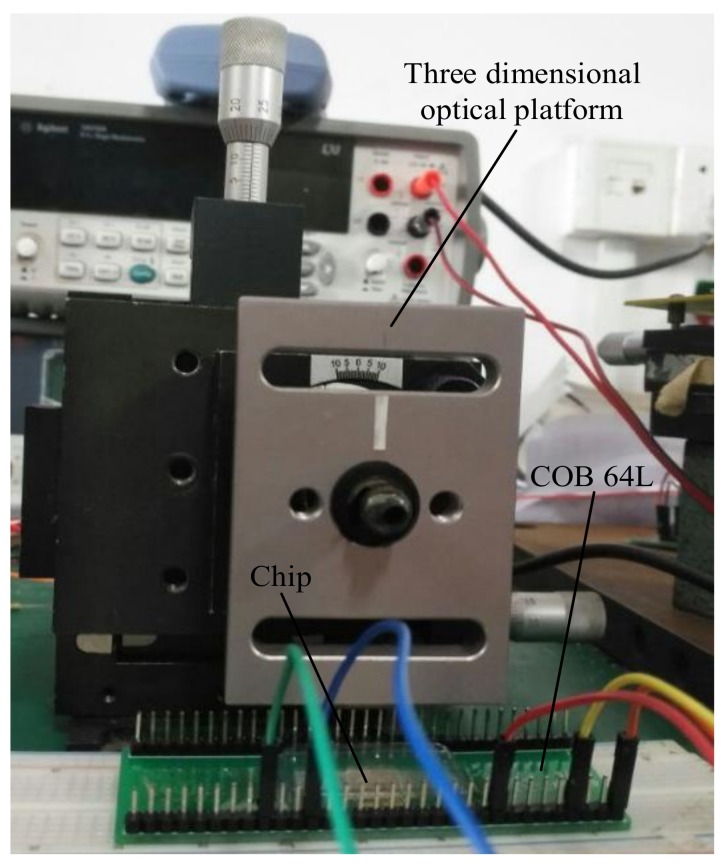
Photo of the experimental platform.

The iOE-PS chip is first glued in a 64-lead chip-on-board package (COB 64L) and wire bonded. Then an 850 nm VCSEL chip is bonded and electrically connected to the pads on the iOE-PS chip. For realizing the proper micromirror position, the MEMS micromirror is placed on a microstage with a displacement accuracy of 0.01 mm and an angle accuracy of 0.1°. Experimental platform is shown as Figure 12.

**Figure 13:**
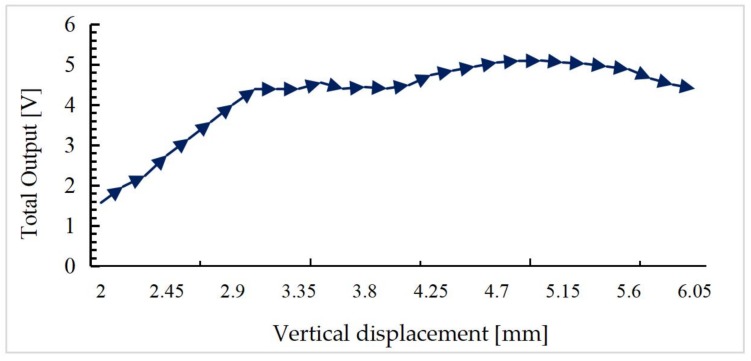
The total voltage versus vertical displacement.

The MEMS mirror is adjusted using the microstage such that the output voltages of the four PDs are equal, which means the mirror plate is parallel to the iOE-PS chip now. Then the mirror plate is moved up step by step using the microstage. At each vertical displacement of the mirror plate, the output voltages of all four PDs are recorded and used for further processing. The sum of the four output voltages is calculated for all positions. The total voltage versus the vertical displacement of the micromirror is plotted in Figure 13, where the total voltage first increases and then becomes slow and decreases. The result of up interval is consistent with the simulation result in Figure 10. In addition, the total voltage increases linearly from 1.5 V to 4.4 V with the displacement between 2.0 mm and 3.0 mm, showing a relatively good linear relationship and a sensitivity of 2.9 V/mm, and reaches its maximum at about 5 mm. The saturation behavior from 3.0 mm to 5.6 mm is due to the saturation behavior of QPD. On one hand, it is because the light power per unit area is also attenuated during the gradual saturation of the optical area. On the other hand, the responsibility of the device itself also impedes the continued increase of the photocurrent at large light power. That is the reason of the low fluctuation of photocurrent in enough light. 

**Figure 14:**
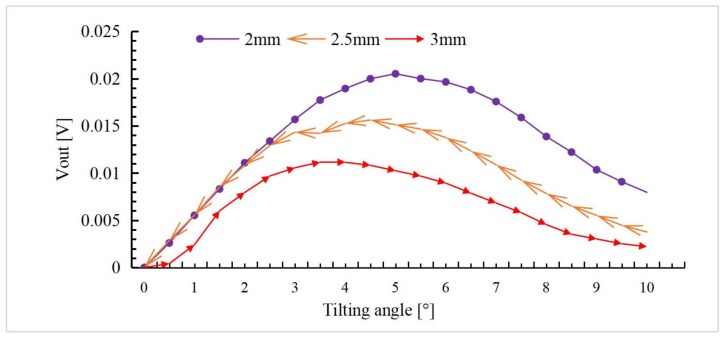
The differential output voltage versus the tilting angle.

To measure the response of the mirror plate tilting, the vertical displacement of them mirror plate is set at 2 mm, 2.5 mm, and 3 mm, respectively. The tilt angle of the mirror plate is swept from 0 to 10° at a step of 0.5° at each vertical displacement position and the output voltages *V_A_*~*V_D_* are recorded for all angles at all positions. To reduce the errors, Δ*V_A_*~Δ*V_D_* are obtained by subtracting each output from the initial value at the tilting angle of 0°. The differential output voltage is calculated using Equation (14). The results are plotted in Figure 14.

(14) $$V_{out}=(\Delta V_{A}+\Delta V_{D})-(\Delta V_{B}+\Delta V_{C}),$$

The differential output voltage first increases with the tilting angle but then decreases, which is consistent with the simulation results in Figure 11. It can be seen that there is also a gentle interval in the middle as a result of the saturation behavior of QPD above mentioned. The drop of output voltage from 5 to 10° is a result that a large dose of light is outside the QPD as tilting angle increases. The linear range spans 0~5° at the vertical displacement of 2 mm, where an angular sensitivity of about 4.2 mV/° is obtained. It can also be seen that there is a larger linear range at a smaller vertical displacement. In a real application scenario, we had better determine the scope of the monitoring before use. If the actual monitoring range is so big that it covers the two monitoring intervals, we can refer to the previous output to judge whether it is in the ascending or descending area or turning area. Generally, the scanning angle range of the micromirror is very small.

## 5. Conclusions

An integrated optoelectronic chip, so-called iOE-PS, has been designed, optimized, and fabricated specifically for position sensing for electrothermal MEMS mirrors. Detailed optical analysis has been performed for determining the wavelength and divergence of the light source, the optimal distance between the MEMS mirror plate and the iOE-PS chip, and the applicable tilting angle range. Experimental results indicate that the optical performant of the iOE-PS chip is optimized by setting the PD spacing to 500 µm, the PD area to 500 × 500 µm^2^, and the light source to the 850 nm VCSEL with a 20° divergence angle. According to the experimental data, this iOE-PS/MEMS module has a linear range over 2.0~3.0 mm for displacement measurement and over −5~5° for tilting angle measurement. With further development of compact packaging, this iOE-PS method will offer large-scan-range electrothermal MEMS mirrors with integrated high-accuracy displacement and angle sensing so that closed-loop feedback control can be readily implemented. This will greatly expand the application space of such electrothermally-actuated MEMS scanning mirrors.
